# Recommended methodologies for clinical investigations of high-risk medical devices—Conclusions from the European Union CORE–MD Project

**DOI:** 10.1016/j.lanepe.2025.101460

**Published:** 2025-09-15

**Authors:** Alan G. Fraser, Sergio Buccheri, Robert A. Byrne, Per Kjaersgaard-Andersen, Stefan James, Peter Jüni, Lia Bally, Richard Bulbulia, Berthold V. Koletzko, Martin J. Landray, Claudia Louati, Anne Lübbeke, Perla J. Marang-van de Mheen, Peter McCulloch, Bernadeta Patro-Golab, Frank E. Rademakers, Petra Schnell-Inderst, George C.M. Siontis, Marina Torre, Claudia Wild, Yasemin Zeisl, Tom Melvin, Rob G.H.H. Nelissen, CORE–MD Consortium, Piotr Szymański, Chris P. Gale, Aldo P. Maggioni, Chris Wilkinson, Fernanda Santana, Valentina Tageo, Jean-Baptiste Rouffet, Adrian Ott, Elizabeth Macintyre, Marieke Meijer, Loredana Simulescu, Kathrin Guerlich, Hannes Jarke, Valentina Strammiello, Lotje A. Hoogervorst, Ewout W. Steyerberg, Jasper W.A. van Egeraat, Alma B. Pedersen, William Plath, Baptiste Vasey, Marion Mafham, Louise Bowman, James A. Smith, Christophe Combescure, Christophe Barea, Jonas Oldgren, Laurna McGovern, J.J. Coughlan, Rory Durand, Stephan Windecker, André Frenk, Bernadette Coles, Arjola Bano, Juri Künzler, Faina Wehrli, Lum Kastrati, Markus Laimer, Elisabetta Biasin, Erik Kamenjasevic, Janos Meszaros, Isabelle Huys, Pascal Borry, Ursula Rochau, Tanja Planinschitz, Uwe Siebert, David M. Epstein, John E. Chaplin, Ola Rolfson, Enrico G. Caiani, Yijun Ren, Donal B. O'Connor, Gearóid McGauran, Gearóid O'Connor, Niall MacAleenan, Sanjeev Yoganathan, Jan Szulc, Robert E. Geertsma, Jantine W.P.M. van Baal, Paola Laricchiuta, Eugenio Carrani, Stefania Ceccarelli, Enrico Ciminello, Sabine Ettinger, Juan A. Blasco-Amaro, Agnieszka Dobrzynska, Jesús Aranda, Juan C. Rejon-Parrilla, Françoise Schlemmer, Sabina Hoekstra, Richard Holborow, Marianna Mastroroberto, Erman Melikyan, Alexey Shiryaev, Christoph Ziskoven

**Affiliations:** a Department of Cardiology, University Hospital of Wales, and School of Medicine, Cardiff University, Heath Park, Cardiff CF14 4XW, UK; b Cardiovascular Imaging and Dynamics, KU Leuven, Leuven, Belgium; c Department of Medical Sciences, Uppsala University Hospital, and Uppsala Clinical Research Center, Uppsala Science Park, Dag Hammarskjölds Väg 38, Uppsala SE-751 85, Sweden; d Cardiovascular Research Institute (CVRI) Dublin and Department of Cardiology, Mater Private Network, Dublin, Ireland; e School of Pharmacy and Biomolecular Sciences, RCSI University, Dublin, Ireland; f Department of Orthopaedic Surgery, Vejle Hospital, South Danish University, Vejle 7100, Denmark; g Clinical Trial Service Unit, Nuffield Department of Population Health, University of Oxford, Oxford OX3 7LF, UK; h Department of Diabetes, Endocrinology, Nutritional Medicine and Metabolism, Inselspital, Bern University Hospital and University of Bern, Freiburgstrasse 15, Bern 3010, Switzerland; i Department of Paediatrics, Dr. von Hauner Children's Hospital, LMU University Hospital, and German Center for Child and Adolescent Health, Lindwurmstr. 4, Munich 80337, Germany; j European Patients Forum, Chaussée d’Etterbeek 180, Brussels 1040, Belgium; k Division of Orthopaedics & Trauma Surgery, Geneva University Hospitals and University of Geneva, Switzerland; l Nuffield Department of Orthopaedics, Rheumatology and Musculoskeletal Sciences, University of Oxford, Oxford, UK; m Safety & Security Science, Delft University of Technology, Jaffalaan 5, 2628 BX, Delft, the Netherlands; n Nuffield Department of Surgical Sciences, University of Oxford, John Radcliffe Hospital, Headington, Oxford OX3 9DU, UK; o Biomedical Sciences, KU Leuven, Herestraat 49, Leuven 3000, Belgium; p Institute of Public Health, Medical Decision Making and Health Technology Assessment, UMIT TIROL – University for Health Sciences and Technology, Eduard-Wallnoefer-Zentrum 1, Hall in Tirol 6060, Austria; q Department of Cardiology, Bern University Hospital, University of Bern, Bern, Switzerland; r Istituto Superiore di Sanità, Viale Regina Elena, 299 00161, Roma, Italy; s Austrian Institute of Health Technology Assessment, Garnisongasse 7/20, Vienna 1090, Austria; t Institute for Clinical Trials, College of Medicine, Nursing and Health Sciences, University of Galway, Ireland; u Department of Orthopaedic Surgery, Leiden University Medical Center, Leiden 2300 RC, the Netherlands

**Keywords:** High-risk medical devices, Trial methodologies, Randomised controlled trials, Registries, Hierarchy of evidence

## Abstract

Before a high-risk medical device is approved for implantation into patients, there should be evidence not only of its performance and safety with a favourable benefit-risk ratio, but also of its clinical efficacy. Regulatory guidance on study methodologies is lacking, however, so the European Commission funded the CORE–MD project (Coordinating Research and Evidence for Medical Devices) to advise regulators on appropriate designs for clinical trials of high-risk devices. The CORE–MD consortium recommends that evaluation should be planned in four stages. Randomised controlled trials should be performed more often, against active comparators reflecting the best available treatment, or using sham interventions with ethical safeguards. Large trials can be managed efficiently using an electronic database or registry. Non-randomised clinical studies can apply objective performance criteria or other validated patient-relevant outcome measures, with adjustments to minimise bias. Full transparency of results from clinical investigations is essential. Proportionate regulation of breakthrough or orphan devices for independently-defined serious unmet needs may involve approval with less evidence, but on condition of subsequent confirmatory studies. These CORE–MD consensus proposals have been submitted to European Union medical device regulators, to be considered as a basis for more transparent and predictable requirements for clinical evidence.

**Funding:**

The CORE–MD project was funded as a Coordination and Support action from the 10.13039/501100000780European Union10.13039/501100007601Horizon 2020 research and innovation programme, under grant agreement 965246.

## Introduction

It is a truism that medical devices are not drugs, so they need to be regulated differently—but from a clinical perspective, the primary objective should be the same whether a new drug or high-risk medical device is being approved. Ideally, there will be robust evidence from well-conducted prospective clinical investigations, showing a beneficial impact on health that outweighs any known risks.

Our CORE–MD consortium (Coordinating Research and Evidence for Medical Devices; www.core-md.eu) was established with the mission of “Translating expert knowledge into advice for EU regulators”. We systematically reviewed 641 studies used to establish clinical evidence for 114 high-risk medical devices: 71 from cardiology and cardiac surgery,^1^ 30 orthopaedic implants,^2^ and 13 systems used in diabetic medicine.^3^ The proportions that were randomised trials were 19%, 9% and 29% for cardiovascular, orthopaedic and diabetes studies; and 9%, 0%, and 18% respectively had been published when the devices were approved. Another CORE–MD review found similar lack of evidence for medical devices used in infants and children.^4^ For orthopaedic devices, the median interval between inclusion of the first patient and the first published study was 10 years, while 13% were registry cohorts with post-market clinical follow-up.^2^ Collectively, these reviews revealed a wide gap between publicly available evidence and the expectations of patients and healthcare professionals that a medical device should be shown to be safe and effective before it is approved.

In a recent study of 6387 adults aged >40 years who participated in the National Health and Nutrition Examination Survey (NHANES) in the USA, 32% had implanted metal devices.^5^ A comparable figure of 30% was reported from a representative population sample of 1400 people surveyed in Hungary.^6^ A majority of high-risk devices are cardiovascular and orthopaedic; an analysis of 379 high-risk therapeutic devices approved through pre-market authorisation by the US Food and Drug Administration (FDA) between 2013 and 2023, found that 50% were cardiological and 10% orthopaedic.^7^ Considering this high prevalence of use, and the dearth of published evidence, there is a need for clear regulatory guidance on what should constitute sufficient high-quality scientific evidence—but current standards refer only to broad general principles.

A call from the European Union (EU) Horizon 2020 research programme stated that the need to generate improved clinical evidence could be addressed “by developing and promoting methodological approaches adapted to the specificities of high-risk medical devices”.^8^ In particular, regulators sought expert advice on establishing a hierarchy of study designs. A principal objective of the CORE–MD project was therefore to review and recommend methodologies that can provide high-quality clinical evidence. These CORE–MD proposals aim to provide manufacturers, assessors in notified bodies, clinicians and patients with greater clarity on the levels of clinical evidence that should be required for the approval of new high-risk medical devices.

## Methods

The objectives and design of the CORE–MD project have been published^9^; it was led by the European Society of Cardiology (ESC) with the European Federation of National Societies of Orthopaedics and Traumatology (EFORT). The study group included leaders of the IDEAL Collaboration (Idea, Development, Exploration, Assessment, Long-term follow-up),^10^ the Good Clinical Trials Collaborative,^11^^,^^12^ the Coalition for Reducing Bureaucracy in Clinical Trials,^13^ the International Society of Arthroplasty Registries,^14^ and EuroHeart.^15^ The composition of the consortium encompassed not only clinical academics and trialists from adult and paediatric medical and surgical specialties, but also experts in statistics, regulatory science, patient-reported outcomes, device registries, and health technology assessment; patients' representatives; and colleagues from European public health authorities, regulatory agencies, and notified bodies. The Advisory Board included international medical device regulators, and physicians from manufacturers’ trade associations.

Medical devices are classified according to the rules stated in Annex VIII of the EU Medical Device Regulation 2017/745 (MDR).^16^ All “implantable devices and long-term surgically invasive devices” apart from those placed in the teeth are designated as high-risk, in Class IIb or Class III (Rules 6 & 8). The primary objective of the CORE–MD project was to recommend methodologies appropriate for the clinical investigation of Class IIb and Class III devices, with a focus on cardiovascular and orthopaedic devices since they have been estimated to constitute up to two thirds of all implanted devices.

Any recommendations for the design and conduct of pivotal clinical investigations of high-risk medical devices were extracted in a systematic review^17^ from 30 regulatory guidance papers, 12 standards from the International Organization for Standardization (ISO), and four consensus statements from academic research consortia.^18^ European medical device regulators and notified body assessors confirmed the lack of standards for evaluating specific high-risk devices, assessing risk-benefit ratios, and defining thresholds for acceptability.^19^ Clinicians serving on EU Expert Panels reported the need for guidance on the advantages and disadvantages of different study designs, including the choice of comparators.

Methodologies used in clinical studies of high-risk devices were documented by systematic reviews. The published protocols give details of search strategies and data extracted concerning cardiovascular^20^ and orthopaedic^21^ implants, devices used in diabetic medicine,^22^ devices for treating children,^23^ studies of patient-reported outcomes^24^ and methodologies used in medical device registries for post-market surveillance.^25^ An overview is provided in Table 1.

**Table 1:** Overview of CORE–MD systematic reviews.

Topic/Protocol & results	Databases searched	Inclusion dates	Number of papers screened	Number of unique studies included
Cardiovascular devices^20^ Siontis et al.^1^	Medline Embase Cochrane Library (CENTRAL)	2000–2021	44,774	264
Orthopaedic devices^21^ Lübbeke et al.^2^	Medline (PubMed) Embase Web of Science Device registry websites and reports	1995–2021	2912	151
Devices for diabetes management^22^ Bano et al.^3^	Medline All (Ovid) Embase (Elsevier) Cochrane Library (Wiley) Science Citation Index Expanded and Emerging Sources Citation Index (Web of Science)	2009–2024	1519	182
Devices for children^23^ Guerlich et al.^4^	Medline (PubMed) Embase	2017–2022	1692	99
Patient-reported outcomes^24^ Rolfson et al.^26^	Medline (PubMed) Cochrane Library (Trials) Cumulative Index to Nursing and Allied Health Literature (CINAHL) clinicaltrials.gov	2000–2022	4138	165
Medical device registries^25^ Hoogervorst et al.^27^	Medline (PubMed) Embase Web of Science Cochrane Central Library Ovid Emcare Google Scholar Centre for Reviews and Dissemination, York University Network of Orthopaedic Registries of Europe (NORE) website International Society of Arthroplasty Registries (ISAR) website	2013–2021	3087	108

A workshop was held at the University of Oxford in November 2022, after a preparatory study undertaken by clinical trialists specialised in medical devices and registries from Uppsala University, together with epidemiological and methodological experts from the Clinical Trial Service Unit and Epidemiological Studies Unit of the University of Oxford; conclusions were published as a report on large simple trials (also known as registry trials or nested studies, and sharing features with pragmatic trials).^28^ A schematic hierarchy of investigations was circulated for comment, then adapted and reviewed at a second workshop held at the BioMedical Alliance in Brussels in November 2023, that was attended by > 40 members of the consortium. There were participants from patients’ associations, notified bodies, EU national regulatory agencies, the Directorate-General for Health and Food Safety (DG SANTE) of the European Commission, and health technology assessment agencies.

The prior systematic reviews^1^, ^2^, ^3^, ^4^ and the CORE–MD recommendations for clinical investigations of medical devices in children^29^ were taken into account. Recommendations from the CORE–MD consortium for the clinical investigation of artificial intelligence- and machine learning-enabled medical devices have been published separately^30^; a recent analysis of devices approved by the FDA reinforces the need for specific standards for their clinical evaluation.^31^

Proposals were discussed with the international Advisory Board of CORE–MD, and with EU medical device regulators at two workshops with the Clinical Investigation and Evaluation Working Group (CIE) of MDCG. The revised recommendations were reviewed with all consortium partners at the last meeting of the CORE–MD Project Board on 14th March 2024, and thereafter edited in a series of conference calls before being circulated for final consensus approval.

## Analysis of existing guidance

**Fig. 1:**
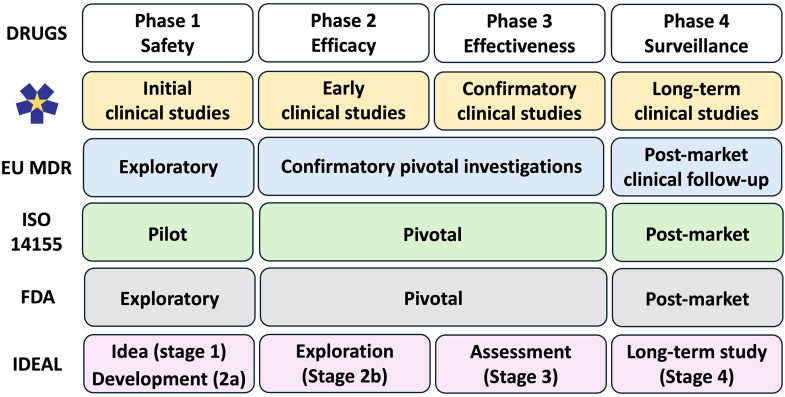
**Comparison of terminologies for stages of clinical evaluation of new drugs and medical devices.** The symbol at the start of the second line is the logo of the CORE–MD project (www.core-md.eu). The other lines summarise the terms used in regulations and guidance: EU MDR, European Union Medical Device Regulation 2017/745^16^; ISO 14155, International Organization for Standardization^34^; FDA, US Food & Drug Administration^32^; IDEAL Collaboration.^10^

General principles were discussed in eight of the 30 regulatory guidance documents.^18^ Four indicated levels of evidence and appropriate clinical investigations for high-risk devices, but only those from the US Food and Drug Administration (FDA)^32^ and the Therapeutic Goods Administration (TGA) of Australia^33^ presented them as a hierarchy (Table 2). In guidance on pivotal clinical investigations for pre-market approval, the FDA listed a choice of study designs, and summarised the phases of clinical assessment as ‘exploratory’, which includes first-in-human and feasibility studies, ‘pivotal’ which evaluates safety and efficacy, and ‘post-market’^32^ (Fig. 1). The TGA in June 2022 referred to a tool for ranking study designs while synthesising evidence, that had been published by the Australian National Health and Medical Research Council (NHMRC) in 2009 (see Table 2).^33^

**Table 2:** Published hierarchies of study designs for medical devices.

US FDA	TGA/NHMRC
	1. Systematic reviews of randomised controlled trials.
**Randomised studies with concurrent controls**	
1. Randomised triple-blinded^a^ parallel-group study with placebo (sham) control. 2. Randomised triple-blinded^a^ parallel-group study with active control. 3. Randomised parallel-group study with incomplete blinding; and active or no-intervention^b^ control group. 4. Randomised study in which patient serves as own control (e.g. split face, cross-over).	2. Individual randomised controlled trials.
**Non-randomised studies with concurrent controls**	
4. Non-randomised study with concurrent controls (explicitly not recommended)^c^	3. Pseudo-randomised controlled trials. 4. Comparative study with concurrent controls (experimental study, cohort study, case–control study, interrupted time series with controls).
**Non-randomised studies with non-concurrent controls**	
5. Single-arm study compared to baseline.^d^ 6. Single-arm study with historical controls with individual patient data. 7. Single-arm study with literature (historical) control.	5 Comparative study without concurrent controls (historical control; two or more single-arm study including unadjusted comparisons; interrupted time series without parallel control).
**Non-comparative studies**	
8. Single-arm study with objective performance criteria or performance goals.	6. Case series.

US **FDA**, United States Food & Drug Administration.^32^

**TGA**, Therapeutic Goods Administration^33^; NHMRC, National Health and Medical Research Council.

a ‘Triple-blinded’ here means blinding of subject, investigator, and third-party evaluator (as recommended by FDA; but note that this term is not used by the FDA).

b A ‘no intervention’ group is not the same as a placebo group; it can be standard of care, or doing nothing.

c A non-randomised study with concurrent controls is explicitly not recommended, because it is as labour-intensive as a randomised study but introduces more biases.

d Single-arm studies compared to base-line are classified as mostly inappropriate.

Governance of the European regulatory system is summarised in the Box 1. Responsibility is shared between national authorities and the European Commission, meeting as the Medical Device Coordination Group (MDCG) which is the statutory body established under Article 103 of the MDR.^16^ Working Groups of MDCG prepare guidance documents. Approval and certification of individual medical devices are delegated to independent commercial organisations known as notified bodies. They employ expert assessors to review the clinical evidence submitted by manufacturers, but their reports are confidential.

Box 1 **Who regulates medical devices in the European Union?**

The **Directorate General for Health and Food Safety** (**DG SANTE**) of the **European Commission** (Unit D3 Medical Devices) is responsible for legislation and policy.

**National Competent Authorities** (regulatory agencies) are responsible for approving the design of pre-market clinical investigations of medical devices, for post-market activities such as receiving incident reports, and for market surveillance of available medical devices.

**Implementation** is overseen by the Medical Device Coordination Group (MDCG) which is chaired by the European Commission with membership from the national regulatory agencies of all member states.

**Notified bodies** are independent certification organisations designated by the EU and listed in the NANDO database∗. They evaluate the evidence that needs to be submitted by manufacturers for all medical devices other than low-risk (Class I) devices. The process is called a ‘conformity assessment’ because notified bodies must determine if the evidence from clinical evaluation conforms with current requirements. If approved, this leads to CE-marking (for European conformity).

**Expert Panels** were introduced by the MDR so that independent clinical specialists can provide opinions on assessments conducted by notified bodies; this is known as a Clinical Evaluation Consultation Procedure. Expert panels also provide advice to some developers of high-risk devices (see Article 61 (2) of the MDR) or orphan devices. Expert panels are managed by the European Medicines Agency.

∗ https://webgate.ec.europa.eu/single-market-compliance-space/notified-bodies.

The MDR states in Annex I on General Safety and Performance Requirements that medical devices “shall be safe and effective”, but otherwise there are references only to “clinical performance”.^16^ Annex XV on Clinical Investigations states (in paragraph 3.6) that the manufacturer will describe the “design of the clinical investigation with evidence of its scientific robustness and validity”. An MDCG guidance document published in 2020 concerns the clinical evaluation of legacy devices (i.e. those approved under the previous regulatory framework); it includes in Appendix III a “Suggested hierarchy of clinical evidence for confirmation of conformity with relevant General Safety and Performance Requirements under the MDR…ranked roughly in order from strongest to weakest”.^35^ Its 12 levels do not describe methodologies, however, with the highest being listed vaguely as “Results of high-quality clinical investigations”, creating a circular argument. Later MDCG guidance on clinical investigations mentions pilot and pivotal investigations but provides no specific examples.^36^

EU guidance does not indicate which particular methodologies should be used to generate the clinical evidence required for certification (equivalent to regulatory approval) of specific device types. The MDR at Article 61 refers to clinical data providing “sufficient” clinical evidence, but what is considered sufficient is unclear. It was intended that common specifications would provide such information, but they have not been developed. Instead, the EU relies on ISO standard 14155, which offered a framework for considering stages of clinical evidence for devices in all risk classes under the previous EU medical device directives.^34^ The ISO document indicates general principles rather than specific study designs.

Medical device legislation in the EU thus leaves responsibility to manufacturers to select the study design that they consider appropriate for their device, and it requires notified bodies to determine if the evidence presented by the manufacturer demonstrates sufficient safety, performance, and “clinical benefit”. EU rules on consultancy prohibit notified bodies from advising manufacturers in advance about the evaluation designs that they would be likely to accept. This contributes to variability of interpretation and unpredictability of regulatory decisions, which reinforces the need for more precise recommendations on study designs.

## Recommendations

A primary requirement for all clinical investigations must be transparency of objectives, methodology, inclusion/exclusion criteria, interventions, comparators, analyses, and outcomes. Studies should conform with the FAIR principles (findable, accessible, interoperable, reusable) for sharing data.^37^ Any hierarchy of designs presupposes that each study will be performed to a high scientific standard, after ethics approval. Every study must be registered in advance, and its protocol including the power calculation and planned statistical analysis published in an open-access database before patients are recruited.

Observational studies including registry cohorts that are intended to acquire data for a regulatory submission should also have a protocol and statistical analysis plan registered, before obtaining and analysing the data. The statistical protocol can describe the estimands, and any necessary protocol changes must be proposed before the data are analysed, and approved only if there is very sound justification.

### Choice of study design

The strength of evidence will be determined by the design of the study, which should be appropriate for the risk class, the stage of development of the device^10^ and its context of use.

The optimal design of a confirmatory (or pivotal) study of relative effectiveness, with the highest internal validity, is a randomised controlled trial (RCT) comparing the device versus a state-of-the-art alternative device or other control and reporting clinical end-points of benefit to patients. The control should be the best proven intervention, but if there is no effective treatment then a placebo may be used (see principle 33 in the Declaration of Helsinki).^38^ Any pharmacological comparator should be the most effective drug available,^39^ given at an appropriate dose. A washout period of non-prognostic medications, that are not essential for the patient because they do not influence major clinical end-points, can be considered so that the ‘isolated treatment effect’ of the device can be tested.

If a placebo effect is suspected as the explanation for an outcome observed in early studies, then a sham intervention must be considered for the control arm in an RCT,^40^ after careful evaluation of ethical implications for patients and with enhanced informed consent.^41^ Ethics approval may hinge on whether or not the risks of the sham intervention are low enough to justify an altruistic gift by those volunteer subjects who are allocated to receive it, since they will not benefit personally. Measures should be taken to minimise risks, such as selecting the least invasive sham intervention, and patients should be fully informed about their potential allocation to a sham intervention in the control arm.

### Statistical considerations

To reach valid conclusions, a trial must be sufficiently powered for the primary outcome measure, with high completeness of follow-up and sufficient duration and size to detect relevant safety signals. It should be designed with at least 80% power at a two-tailed significance level of p < 0.05 to detect a clinically meaningful effect on a patient-relevant outcome.^42^ The sample size required to detect such an effect will depend, among other factors, on the anticipated event rate in the control group (for time-to-event studies or binary outcomes such as failure of a device, hospital admission, or death) and/or the precision of the outcome measure (for continuous variables such as blood pressure, glycated haemoglobin, or quality of life). If the planned primary outcome is a surrogate endpoint, then the practical or scientific reasons for using it must be justified. The power calculation will also be influenced greatly by the anticipated effect size of the intervention. An expert task force which advised EU regulators on clinical investigations of coronary stents, estimated that studies reporting surrogate outcomes could have a sample size of 150–300 patients, while studies powered for clinical endpoints would typically enrol 1500–3000 patients.^43^ An adaptive trial design can allow investigators to modify their trial's conduct, increasing its utility while retaining its integrity.^44^

When a hypothesis of superiority is not plausible, a non-inferiority or equivalence RCT that applies realistic assumptions about margins may be acceptable. The assumed margins should be supported by evidence. Most non-inferiority trials are powered using absolute non-inferiority margins on a risk difference or a difference in cumulative incidence scale, as this allows for smaller sample sizes compared with trials powered under assumptions on a relative scale (hazard ratio, risk ratio, rate ratio, or odds ratio). Guidance from the European Medicines Agency in 2005, concerning non-inferiority margins in drug studies, advised that the margin should be independent of considerations of power and supported by clinical arguments for a meaningful difference.^45^ It should take account of historical evidence about the active comparator.^46^ In a systematic review of drug studies, the overall non-inferiority margin was an absolute risk difference of 9% (IQR 4.2%–10%).^47^ An absolute margin of 9% would be too large, however, if the control group event rate is below 30%; applying that standard would run the risk of falsely concluding that new procedures are as safe and effective as those in current use.

For device studies, we recommend that results should ultimately be interpreted on the 95% confidence interval of a relative effect measure (e.g. hazard ratio) rather than on an absolute scale.^48^ The consensus recommendation of a methodology task force of the ESC Clinical Practice Guidelines Committee was that a tested intervention can be considered to meet non-inferiority if it has been demonstrated that it is unlikely to be more than 15% worse than the control intervention for the outcome of interest, using a relative measure; in that case, the upper limit of a two-sided 95% confidence interval for a relative effect measure should fall below 1.15, when assessing an unfavourable outcome such as device failure.^48^ The standard error for a trial reaching this relative margin with the upper limit of the 95% CI is comparable to the average standard error found when detecting a hazard ratio of 0.8 at P < 0.005 with 80% power. Acceptable limits for device trials need to be evaluated more thoroughly.

### Phases of clinical investigations

There is overlap rather than uniformity between the descriptors used by different regulators (see Fig. 1). An earlier EU study group identified four stages of evidence during the lifecycle of a device, but only two were clinical (named pre-market and post-market).^49^ Our CORE–MD consortium now proposes a progression through four stages of clinical investigations for medical devices, from ‘initial’ to ‘early’ to ‘confirmatory’ and then to ‘long-term’ studies. The four stages correspond most closely with recommendations from the IDEAL Collaboration.^10^

**Fig. 2:**
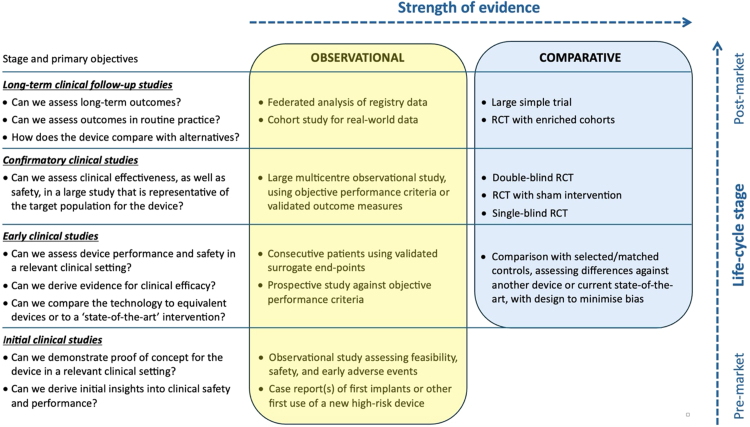
**CORE–MD recommended study designs for high-risk medical devices**. RCT randomised controlled trial. The most reliable evidence comes from experimental studies that compare a device against an appropriate control intervention in an RCT, as shown in the top two boxes in the “Comparative” column. Observational studies that analyse performance of a device against other treatments, or that describe results from a prospective cohort study, will provide supportive rather than definitive evidence.

For pharmaceutical products there is consensus that three phases of clinical trials should be completed before a new drug is licensed. Our scheme has synergism with those phases (see Fig. 1), but since it is difficult to gauge a single step at which regulatory submission would be appropriate for all devices, it also offers flexibility. Thus a major reason for our proposed four-stage framework, rather than the three stated in most guidance documents, is that its stages can be translated into varying levels of evidence appropriate for different devices to be approved in different circumstances, as discussed below. Fig. 2 gives examples of objectives and recommended study designs at each stage.

For a new implantable device, the initial phase of clinical evaluation will have been preceded by iterative development of prototypes, and by pre-clinical investigations such as bench tests, animal studies, and toxicological tests. Computational simulations, for example of the fluid dynamics of a heart valve or the mechanical stresses and strains of an orthopaedic implant, may be particularly informative, but no digital model is a complete reflection of the human body and there are many questions about how their results can be validated.^50^ *In silico* ‘trials’ cannot be considered as a substitute for clinical investigations; they are approaches for estimating pre-clinical evidence and therefore out of scope for our report. Knowledge derived from non-clinical testing and from a systematic analysis of alternative treatments and prior clinical studies helps to refine the research hypothesis relative to the stage of development of the technology.

For the third phase of clinical investigations, the descriptor “confirmatory” is preferred over “pivotal”. Annex XIV of the MDR, on Clinical Evaluation, refers in paragraph 1 to “progression from exploratory investigations, such as first-in-man studies, feasibility and pilot studies, to confirmatory investigations, such as pivotal clinical investigations”,^16^ but the MDR does not define a pivotal investigation. That term is commonly used to describe a study that may provide crucial evidence for regulatory approval, but its broad application to all pre-market investigations beyond the exploratory stage does not distinguish between lower- and higher-risk devices. All studies may be ‘confirmatory’, but here we employ the term to refer to studies designed to provide definitive evidence.

In any confirmatory study, a high proportion of patients should be recruited within the population in which the approved device will be used. The CORE–MD systematic reviews found acceptable inclusion in device studies of both men and women, but little reporting of ethnicity and few analyses of outcomes stratified by age and sex.^51^ Clinical follow-up studies of high-risk devices should also be representative of all patients and comprehensive; the target for inclusion in medical device registries is >95% of all eligible patients.^52^ Follow-up studies should assess the clinical effectiveness of devices according to their intended medical purpose, as well as their safety. Long-term follow-up should continue for the expected lifetime of the device, to detect any late adverse effects. For example, loosening of an orthopaedic implant may become evident only after more than 10 years.

The terms “prospective” and “retrospective” refer to the manner and timing of collecting data rather than to any specific study design, and they are applied inconsistently.^53^ Use of those descriptors has therefore been limited, in our tables. A prospective study typically involves collecting data for a predefined purpose, allowing researchers to control the process and to document potential confounding factors systematically. A retrospective study typically relies on existing data, which may be limited if relevant confounders were not recorded. A cohort study may be retrospective (using existing data to define a cohort and outcomes) or prospective (collecting new data and thus having more control over potential bias).

### Methodologies for clinical studies of high-risk devices

Methodologies that can provide scientifically valid results at different stages are shown in Table 3, approximately in descending order of the quality of evidence that they can generate. The lists distil multiple variants into a few main categories. The CORE–MD consortium strongly recommends that the highest possible quality of evidence should be obtained for any new high-risk medical device before it is approved. Ideally that would mean a randomised controlled trial, but it is recognised that RCTs of devices are not always feasible. In a German study, 57% of 122 applications for ethical approval of studies across a wide range of devices were for RCTs.^54^ Devices should be tested against the best alternative treatment or, when appropriate, a sham intervention.^55^ Validated end-points and clinical events should be determined independently by blinded assessors.

**Table 3:** CORE–MD Recommended study designs and methodologies for high-risk medical devices.

Initial clinical studies	Early clinical studies	Confirmatory clinical studies	Long-term clinical studies
***First-in-human and preliminary clinical studies:*** ***All experience to be publicly reported.*** • Case report(s) of first implants or other first use of a new high-risk device.^a^ • Observational studies assessing feasibility, safety, and early adverse events.^b^	***Assessment of performance, safety, and positive benefit–risk ratio:*** ***Preliminary assessment of efficacy.*** ***Analysis of learning curves.*** • Non-randomised clinical study of intervention (e.g. single-arm, enrolling consecutive patients), using patient-relevant outcomes and/or validated surrogate end-points. • Observational study testing against objective performance criteria (OPC). • Cohort with matched controls, assessing differences against another device or current state-of-the-art.	***Further demonstration of safety*.** ***Assessment of efficacy for clinical outcomes***^c^***:*** • Double-blind RCT, if feasible. • Single-blind RCT against active comparator^d^ – powered for ‘superiority’.^e^ • ‘Assessor-blinded’ RCT with sham intervention (if no active comparator available, and a placebo effect is suspected).^f^ • Single-blinded RCT (as above) – powered for non-inferiority. • Large multicentre observational cohort, using OPC or other validated outcome measures.	***Long-term monitoring of device performance and safety, in comparison against alternatives:*** • ‘Large simple’ RCT such as a registry-based trial.^g^ • RCT in enriched cohorts.^h^ • Observational cohort using a registry or other real-world source of data, including all devices of the same type, and with results combined through a federated analysis, using appropriate adjustments to minimise bias.

RCT, randomised controlled trial; OPC, objective performance criteria.

The objectives for studies during each stage are indicated by the text in italics at the top of each column

a When use of a new device has been approved for each patient on the basis of an individual humanitarian exemption, data collected retrospectively as a “compassionate-use case series” should not be considered sufficient for regulatory approval of that device.

b Clinical performance and outcomes documented prospectively in a consecutive series of all patients receiving the device, with a pre-planned common protocol for the method of delivery/use of the device and for all data collection, which may be modified iteratively, if necessary, in response to initial experience and outcomes.

c Against active comparator, ‘state-of-the-art’ alternative device, or best alternative treatment; and with (co-determined) patient-relevant end-points.

d Another device already approved for the same clinical indication, whether of similar or alternative design.

e ‘Superiority’ refers to a study designed to accept or reject a null hypothesis of no difference (with a two-sided test for significance).

f ‘Assessor-blinded’ means with independent ascertainment of end-points by blinded observers.

g Large trial with minimal exclusion criteria, and with its conduct simplified by embedding study within electronic health record or similar; encompasses registry trials, nested trials, platform trials, etc.

h If required as a condition on the certificate of conformity, or if a device has been approved with limited evidence because it addresses an important unmet clinical need.

Embedding a trial within a medical device registry or other electronic platform can accelerate recruitment, increase efficiency, and reduce costs.^28^^,^^56^^,^^57^ Large simple RCTs should be encouraged but they may be feasible only once the design of a new high-risk device has become stable or mature^28^; well-designed, properly powered registry-based RCTs should be preferred for confirmatory studies. The term ‘large simple’ trials is preferred over ‘pragmatic’ trials, since all studies should be designed to provide evidence that will be relevant in routine practice.

If a trial is open-label there can be bias in the ascertainment, assessment, and reporting of outcomes particularly if they are subjective. An open-label trial that is underpowered, short in duration, has a high cross-over rate and substantial loss to follow-up, will be poor-quality and unreliable even if it is randomised and controlled. Well-designed and properly powered observational cohort studies can be informative if they have long and complete follow-up, independent adjudication of events, and control of possible confounding factors.

Blinding of independent assessors of clinical outcomes will always be possible and should be the norm. One option is a PROBE design (Prospective randomized open blinded end-point).^58^ Whether studies are experimental RCTs or observational real-world cohort studies, any potential bias should be minimised by the design and/or the analysis (internal validity), so that its conclusions can be generalised to an unselected population (external validity).

Tables 4 and 5 illustrate how this CORE–MD consensus on optimal methodologies for the design and conduct of clinical investigations could align with regulatory submissions in two particular circumstances. Market approval may be considered at different phases in the development of clinical evidence, either when there is uncertainty of evidence (Table 4) or when there is known clinical benefit from similar devices (Table 5).

**Table 4:** Recommended designs for clinical investigations of a breakthrough or orphan high-risk medical device.

If a technology has been designated as a breakthrough or orphan device, then approval with an EU certificate of conformity/CE mark could be considered after the phase of early clinical investigations, and awarded with conditions for the subsequent collection of definitive evidence and with continuing market access thereafter being dependent on confirmation of positive clinical impact.

**Table 5:** Recommended designs for clinical investigations of a new high-risk medical device, with existing options.

Regulatory approval (with the award of an EU certificate of conformity/CE mark) of a new high-risk medical device that is intended for an existing market, implying that other devices have already been approved and are available for the same clinical indication, should normally be considered after evidence has been collected from confirmatory clinical investigations.

^a^Examples could be: for an orthopaedic implant, radiostereometric analysis (RSA) of migration of the device,^59^ and for vascular stents and scaffolds, vessel patency determined using intravascular imaging and objective performance criteria.^43^

^b^Another device of similar type already approved for the same indication.

^c^Recommended only if the manufacturer offers a well-justified explanation for the lack of access to an active comparator for their clinical study, and if a placebo effect is strongly suspected. The least invasive placebo intervention and other risk minimisation measures for patients should apply.

^d^If no active comparator or sham intervention is available, then this table does not apply.

### Orphan medical devices

When a device represents a genuine breakthrough for a condition for which there is no effective alternative device or treatment, or it is intended to treat an orphan disease or indication, then any approval based on limited evidence should be balanced by mandatory post-market clinical studies (Table 4). Three prior conditions would all need to be satisfied: independent experts and patients (unconnected with the development of the device) should have confirmed an unmet need; the intended use should be to diagnose or treat a serious disease or condition; and no alternative devices should already have been approved for the same indication.

It is recommended that devices should not be compared against historical controls, with the exception that it would be ethical to compare a new life-saving device against prior outcomes in patients for whom no therapeutic options were available. Early approval without robust clinical evidence can be considered for a device that is needed by infants and children, if it is impossible to perform an adequately powered prospective study.^29^ Experts can advise regulators about expected benefits and risks, and patients or their families should be involved in ethics decisions about study designs. Patients will want to decide for themselves whether or not to receive a new device, depending on their understanding and perception of the balance between benefits and risks.

### Well-established device types

We recommend that new devices that are intended for clinical indications for which alternative therapeutic options are already available, should normally be approved after the confirmatory stage. Higher standards of clinical evidence should be expected by regulators before approval of a new high-risk medical device that is intended to treat a condition for which alternative devices have already been approved (Table 5). This would apply when the new device is in the same risk class and generic device group according to the European Medical Device Nomenclature, as existing devices, and when the manufacturer does not claim equivalence to any predicate device.

The new device should then be approved only after it has been confirmed to be at least as safe and effective as an existing device or drug(s) representing the ‘state of the art’, in a head-to-head comparison. The CORE–MD consortium considers that the ethical and methodological arguments for expecting the default trial design to be an RCT are convincing. Political and commercial pressures may make this difficult in the short term, but it should remain the long-term goal.

## Discussion

Appropriate use of high-risk medical devices is now essential for the optimal care of many patients with non-communicable diseases such as cardiovascular or orthopaedic conditions or diabetes mellitus. Key regulatory challenges in the EU have included limited transparency of clinical evidence^60^ and variations in the amount and type of clinical evidence required before devices are approved. The MDR was adopted in 2017 but there are concerns that the increased requirements that were introduced have not resulted in efficient regulation. One reason may be that the expert workforce that was anticipated to manage the new system was never provided, but another could be that there has never been a fundamental consideration of the clinical principles that should underpin any regulatory system so that it can deliver proportionate approvals within an evidence-based framework.^61^

The current standard of “clinical data providing sufficient clinical evidence” is subjective, so it remains possible that a high-risk medical device may be marketed in Europe without any pre-market clinical investigation. There remains a need for a clear framework of requirements for clinical evidence, which in an ideal world would include a minimum of at least one large confirmatory RCT.

The consensus view from the CORE-MD project is that no single hierarchy of study designs can be proposed for all high-risk medical devices. Clinical investigations should be structured in four stages, with regulatory submission and review at different time-points depending on the risk class of a device and the clinical need for the device. The systematic reviews performed by the CORE–MD consortium^1^, ^2^, ^3^, ^4^ have shown that there is a greater need for more evidence, and for higher-quality evidence, than for new methodological approaches.

Devices with lower relative risk profiles could be approved at an earlier stage in their lifecycle, once evidence confirms performance, safety and stability,^62^ thereby reducing the administrative burden on regulatory review processes. For high-risk devices, regulatory science should focus on defining which criteria are essential to demonstrate safety before patients receive devices intended to improve clinical outcomes. The preferred study designs mentioned in this report should not be seen as mutually exclusive, and the lists are not exhaustive. Ideally, harmonised standards for trial designs for approval of each major device type would be established globally.

Many high-risk devices have gained market approval without having undergone a confirmatory or pivotal study. Approving devices on the basis of early clinical evidence risks the later occurrence and delayed recognition of problems. Orthopaedic studies of hip and knee implants have shown that a new variant with seemingly small changes may have worse performance than prior devices with the same brand name (causing a phenomenon that has been called camouflage).^63^^,^^64^ When a manufacturer seeks approval for an iterative modification of an existing design, it must satisfy the conditions for equivalence specified in the MDR at Annex XIV (Part 1, paragraph 3)^16^; otherwise new clinical investigations should be performed.

Provisionally accepting certain devices subject to further evidence development is a rational approach that allows the regulator to withhold final approval until a pivotal study has been done; an example would be the ‘Early Feasibility Study’ pathway of the FDA.^65^ The EU regulatory framework, however, does not provide for conditional approval. Instead, notified bodies can impose conditions on certificates, but another CORE–MD study showed that they rarely do so.^66^ Shorter routes to regulatory approval should be reserved for special circumstances. Requiring traditional RCTs for new devices entering existing markets is ethically justified; otherwise, manufacturers would risk introducing a device that is less safe or effective than existing alternatives.

A common criticism is that regulation impedes innovation, but whether or not a medical device is truly ‘innovative’ needs to be determined independently since developers and trialists may have a vested interest. It has been suggested that using the term innovation is unhelpful because it may be assumed to imply that the development is beneficial.^67^ An alternative approach would determine what investigations are necessary, by considering simply if a device represents a genuine technological advance (or breakthrough) and if its benefits and risks are sufficiently understood.

Flexible approval processes can be implemented for unmet needs, if there are universal regulatory standards and full transparency of clinical evidence. Alternatives to RCTs may be considered.^68^ Rigorous adherence to optimal methodologies for observational studies can ensure that they contribute appropriate evidence for regulatory approval. Recent reports have advised how surrogate end-points should be developed, implemented, and reported.^59^^,^^69^, ^70^, ^71^, ^72^ There could be greater use of objective performance criteria (OPC) as a benchmark against which new devices can be compared^73^ provided that the OPC are supported by strong evidence. As an exemplar, a dual process to evaluate evidence for coronary stents was recommended to the European Commission in 2015, applying OPC during the early stage of evaluation and requiring an RCT during the confirmatory stage.^43^

The fourth stage of clinical evaluation, long-term clinical follow-up, may require a change of culture to ensure that all patients with high-risk devices are enrolled in registries.^27^ The CORE–MD investigators support the concept that this should be mandatory when a device has been approved as an orphan product or breakthrough technology for which there is limited evidence. Similar to trial populations, registry cohorts should have wide geographical coverage^74^ and be representative of all patients who may receive the device. Cohorts may be enriched by selecting patients with specific characteristics if there are signals suggesting that they may influence safety or effectiveness.

### Implementation of these recommendations

Current EU guidance for the clinical evaluation of medical devices^75^ dates from 2016 when the medical device directives were still the legal basis for approvals. That guidance did not indicate any specific requirements for particular types of devices nor did it rank study designs in order of their scientific merit. New MDCG guidance on clinical evaluation, applicable to the provisions of the MDR, has been prepared and is now being revised after consultation with stakeholders. These recommendations from the CORE–MD consortium will be considered by a task force of EU medical device regulators, to determine if and how they may inform the further development of European guidance. They have also informed policy on the further reform of the EU regulatory system, published on behalf of medical associations by the Biomedical Alliance in Europe.^76^ Consideration of the EU Health Technology Assessment (HTA) Regulation was not within the scope of the CORE–MD project but high-quality clinical evidence collected as the basis of a submission for regulatory approval of a new device would be suitable also for consideration during HTA joint assessments.

This project was conducted to support the EU framework for medical devices but all major jurisdictions and the World Health Organization support the concept of global regulatory convergence, with mutual reliance on approvals of devices. Beyond the EU, another ISO standard on the clinical investigation of medical devices is under development, and working groups within the International Medical Device Regulators Forum (IMDRF) are considering recommendations for particular classes or types of devices such as those incorporating softwares that use artificial intelligence.

## Conclusions

The clinical evidence of safety and efficacy for many high-risk medical devices needs to be strengthened, particularly by conducting more randomised trials. Clear guidance and technical specifications would make it easier to perform appropriate trials for a specific device. Processes for approving study designs and for monitoring the conduct of studies could be simplified without compromising on their quality. Life-cycle evidence needs to be strengthened through observations of real-world performance and safety, collected from high-quality cohort studies and registries. Regulators should be enabled to engage with trialists and developers in order to provide scientific advice on recommended methodologies.

## Contributors

AGF obtained funding for the CORE-MD project, and drafted this manuscript. SB, SJ, PJ, RB, MJL, and PMcC from the Universities of Uppsala & Oxford contributed to the preparatory manuscript and drafting of a hierarchy of methodologies. The systematic review of guidance was performed by PSI. All authors participated in the workshops and/or on-line discussions to prepare these proposals. All authors reviewed and edited the text, and approved the final version of the manuscript.

## Declaration of interests

AGF chaired the Regulatory Affairs Committee of the Biomedical Alliance in Europe, and was scientific coordinator of the CORE-MD project.

SB has received support for travel from Abbott, paid indirectly to his institution.

RAB does not accept personal payments from manufacturers of medical devices or from pharmaceutical companies; he reports research or educational grants to the institutions of employment that do not impact in any way on personal remuneration, from Abbott Vascular, Biosensors, Boston Scientific and Translumina.

PKA chairs the Medical Device Task Force of the Biomedical Alliance in Europe. He is a member of the EU Expert Panels for Medical Devices and chair of the Screening Panel for Orthopaedics, Traumatology, Rehabilitation and Rheumatology.

SJ has received personal support from Medtronic for valve proctoring. His institution has received research grants from Amgen, Astra Zeneca, MSD, Medtronic, Edwards and Elixir.

LB has received research and product support from Dexcom, Novo Nordisk and Ypsomed; has participated in advisory groups for Dexcom, Roche Diabetes, Sanofi, Novo Nordisk, Eli Lilly; and has received fees for speaking from Dexcom, Eli Lilly and Ypsomed.

RB is a member of the UK MHRA Interim Devices Working Group.

BVK has not received any personal or institutional financial support from the medical device sector. He was a task leader in the CORE MD project, is a member of the Medical Device Task Force of the Biomedical Alliance in Europe, and is President of the European Academy of Paediatrics. His position as Senior Professor of Paediatrics at the University of Munich is supported financially by the charitable Else Kröner-Fresenius-Foundation.

ML is Advisor (for Clinical Trials) to the Regulatory Affairs Committee of the European Society of Cardiology.

AL does not accept personal payments from manufacturers of medical devices or from pharmaceutical companies; she is the current president of the International Society of Arthroplasty Registries (ISAR).

PMcC is chair of the IDEAL Collaboration.

BPG received a grant from the Alexander von Humboldt Foundation, Bonn, Germany.

FER led the task on artificial intelligence/medical device software in the CORE-MD project.

TM is a member of the EU Expert Panels for medical devices; he was paid for providing clinical training to the National Standards Authority of Ireland and the European Commission.

RGHHN is chair of the EU thematic Expert Panel for Orthopaedics, Traumatology, Rehabilitation and Rheumatology. He is cofounder of the Dutch Arthroplasty Registry (LROI).

All other authors have nothing to declare.
